# Supplementary material for: Anti-correlation of LacI association and dissociation rates observed in living cells
Source: Nat Commun. 2025 Jan 17;16:764. doi: 10.1038/s41467-025-56053-z (PMC11748676; doi:10.1038/s41467-025-56053-z)
Supplement: Supplementary file 1 — Supplementary Information [file 41467_2025_56053_MOESM1_ESM.pdf]

Supplementary Information

Anti-correlation of LacI association and dissociation rates observed in living cells.

Supplementary Fig.1

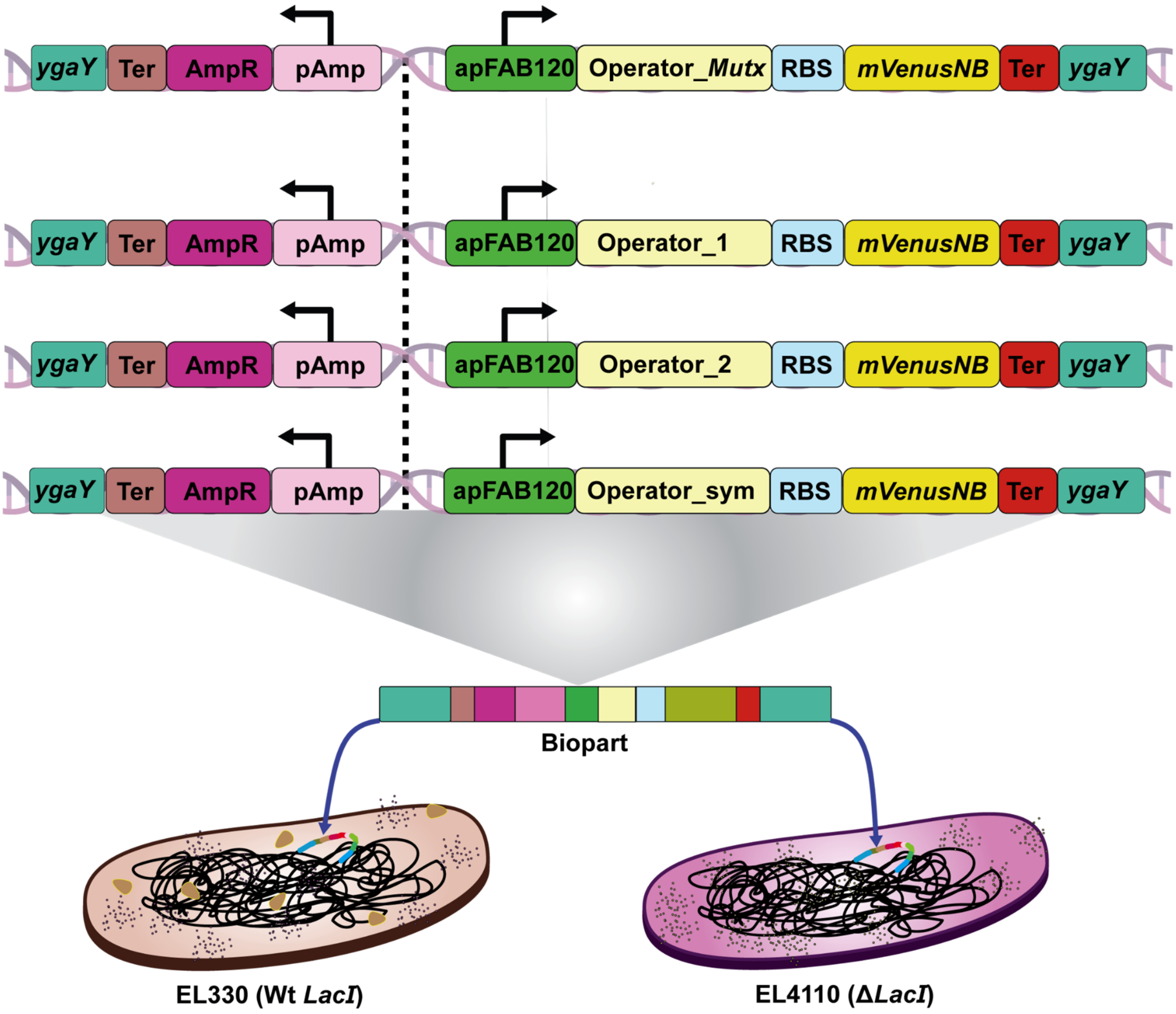

Supplementary Fig. 1: Schematic of promoter-operator Biopart and its insertion into the chromosome. The Biopart encodes the mVenusNB fluorescent protein expressed from the constitutive apFAB120 promoter. Downstream of the promoter, various LacI binding site ("Operator") mutants were introduced. Upstream of this promoter is a gene encoding ampicillin resistance, serving as a selection marker. This biopart is inserted into the *ygaY* locus in the chromosomes of two different host strains, which differ in the presence/absence of the *lacI* gene. This figure is created in BioRender. https://BioRender.com/y55z427.

Supplementary Fig. 2

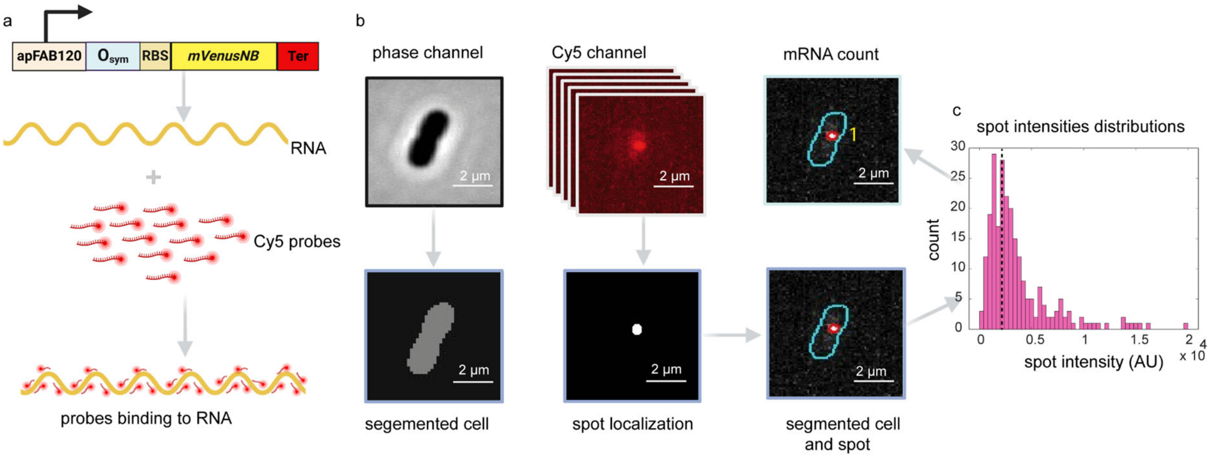

Supplementary Fig. 2: Quantification of single mRNA spot intensities. **a** Illustration of Biopart transcription and binding of Cy5 probes to mVenusNB mRNA (created in BioRender, https://BioRender.com/p79t124). **b** Image acquisition of cells in the phase channel and fluorescence channel (top), cell segmentation, spot localization and segmentation from the Z stacks fluorescence images were performed using the custom-built pipeline in our lab (bottom). **c** Distributions of mRNA spot intensities from observed cells (N=371). The dotted line represents the inferred single mRNA spot intensity value (≈2400 A.U).

Supplementary Fig. 3

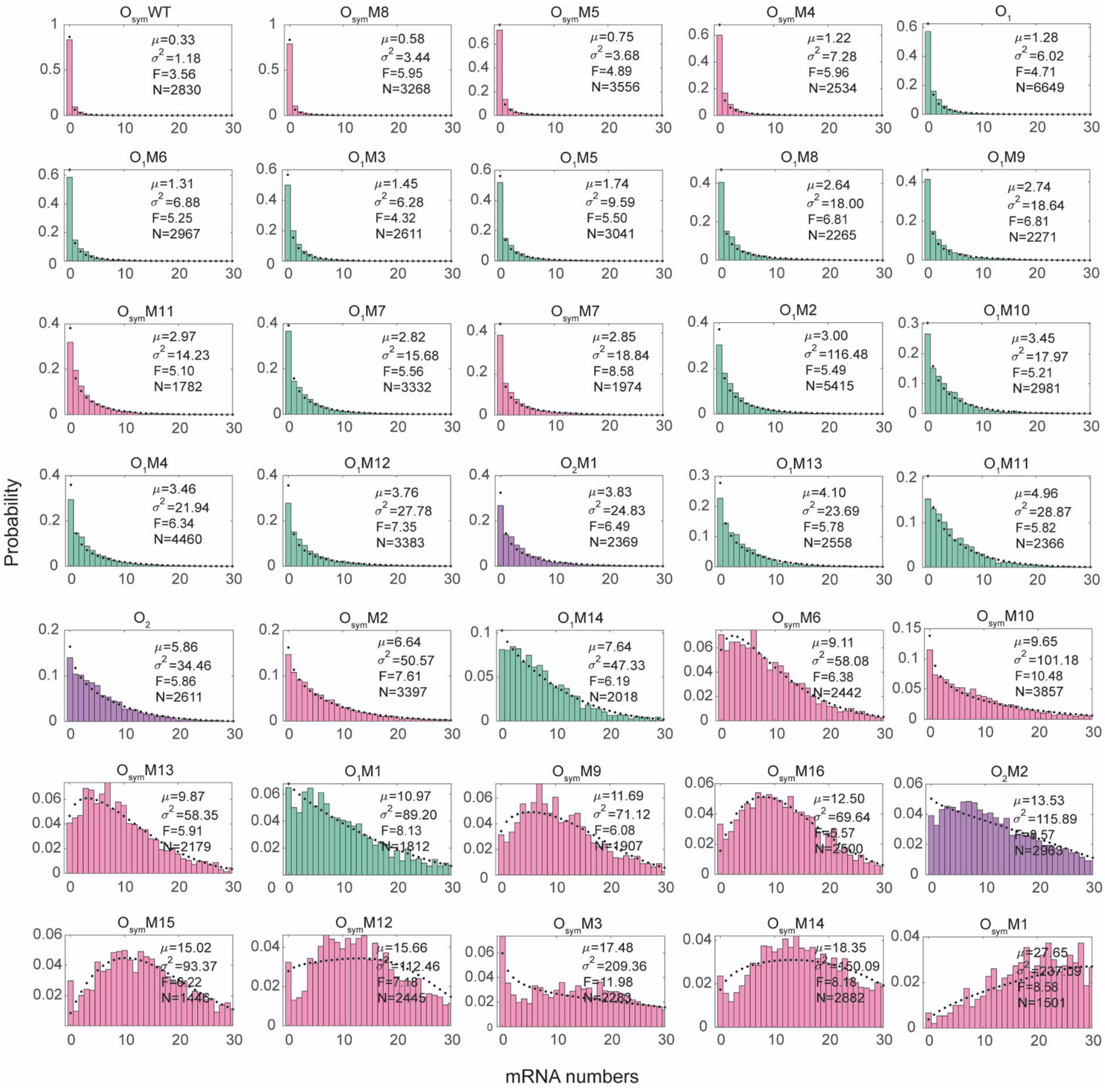

Supplementary Fig. 3: Observed mRNA copy number distributions in *wt lacI* strains, for all operators assayed in this work. For each operator, the probability mass function (pmf) corresponding to inferred r, k_a, and k_d values is indicated with a dotted line.

Supplementary Fig.4

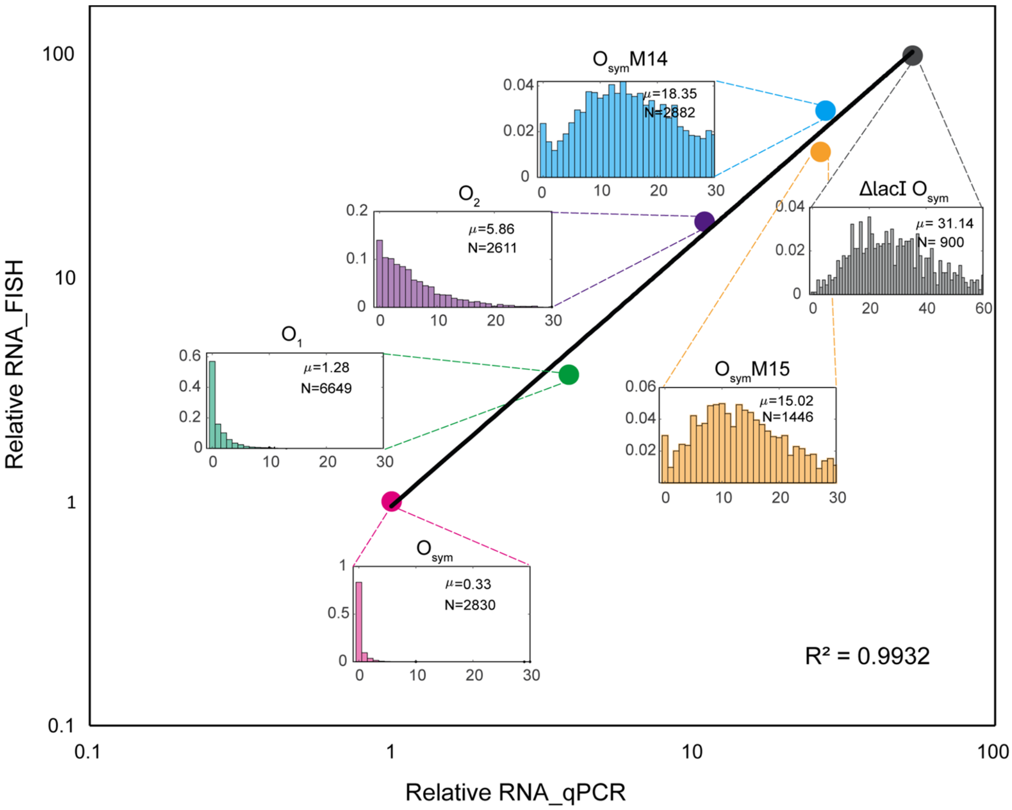

Supplementary Fig. 4: Comparison of mean mRNA FISH data with qPCR measurements. We selected six constructs ranging from low (0.33 mRNA/cell) to high (31.4 mRNA/cell) expression and compared mRNA FISH values with relative qPCR data. We find a positive correlation between these two independent measurements with an R^2 value of 0.9932.

Supplementary Fig. 5

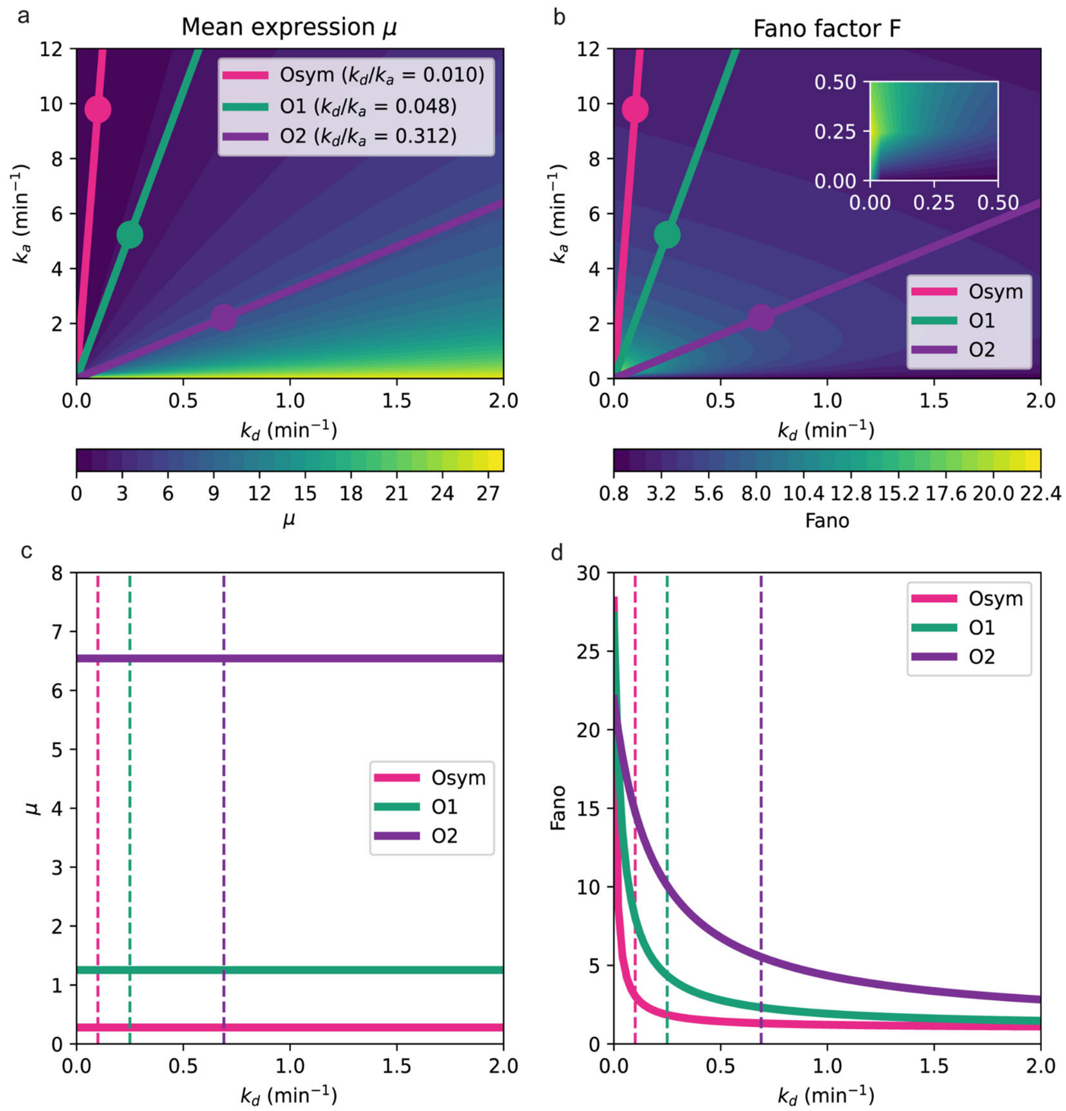

Supplementary Fig. 5: Dependance of mean expression μ and Fano factor F on k_a and k_d, computed from Eqs. 1 and 2. r = 22 min^{-1} and γ = 0.8 min^{-1} for all panels; note that for actual fitting of k_a and k_d in Figure 3, r is measured separately for each operator. Here we choose a "compromise" value of r in order to show all of Osym, O1, and O2 on the same plot for illustrative purposes, meaning that u and F do not perfectly match experimentally measured values for the three operators. **a** Mean expression as a function of k_a and k_d depends only on the ratio k_a/k_d. The mean expression and k_a/k_d ratio for Osym, O2 and O2 are plotted in magenta, green, and purple (respectively). This plot demonstrates how measuring mean expression allows determination of the k_a/k_d ratio, but not their magnitude. **b** Fano factor as a function of k_a and k_d, showing a generally decreasing trend for larger values of k_a and k_d. Inset shows F in the immediate vicinity of the origin. **c** Mean expression plotted as a function of k_d along each of the lines indicated in panel a. As expected, the mean does not depend on the value of k_d given fixed k_a/k_d. Dashed lines indicate experimentally-determined k_d values for each operator. **d** Fano factor as a function of k_d plotted along the lines of constant k_a/k_d for each of the three operators, showing a monotonic decrease from k_d = 0. k_d is determined as the point where the F vs k_d curves intercept the experimentally-measured (corrected) Fano factor values, whereupon k_a is also known because the k_a/k_d ratio is known from the mean expression. A little algebra reveals that the limiting values of F are 1 as $k_d \to \infty$, and $1 + 1/(1+ k_d/k_a)*r/\gamma$ as $k_d \to 0$ for a given k_a/k_d ratio. As long as F lies within these limits, and $0 < \mu < r/\gamma$ (*i.e.*, the operator is occupied between 0 and 100% of the time), Eqs. 1 and 2 will have a unique mathematical solution for k_a and k_d for given values of r and γ.

Supplementary Fig. 6

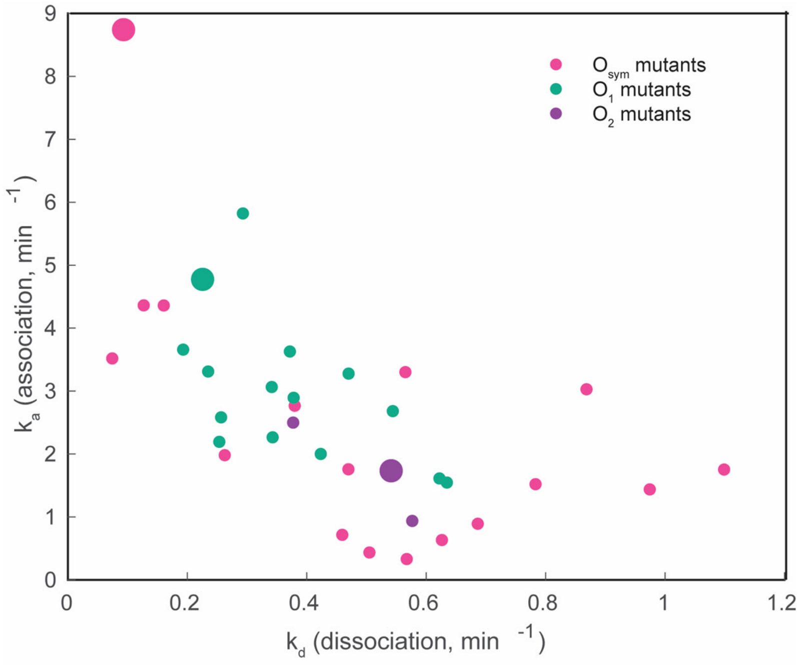

Supplementary Fig. 6: Inferred k_a and k_d values for all 35 operators without Fano factor correction.

Supplementary Fig. 7

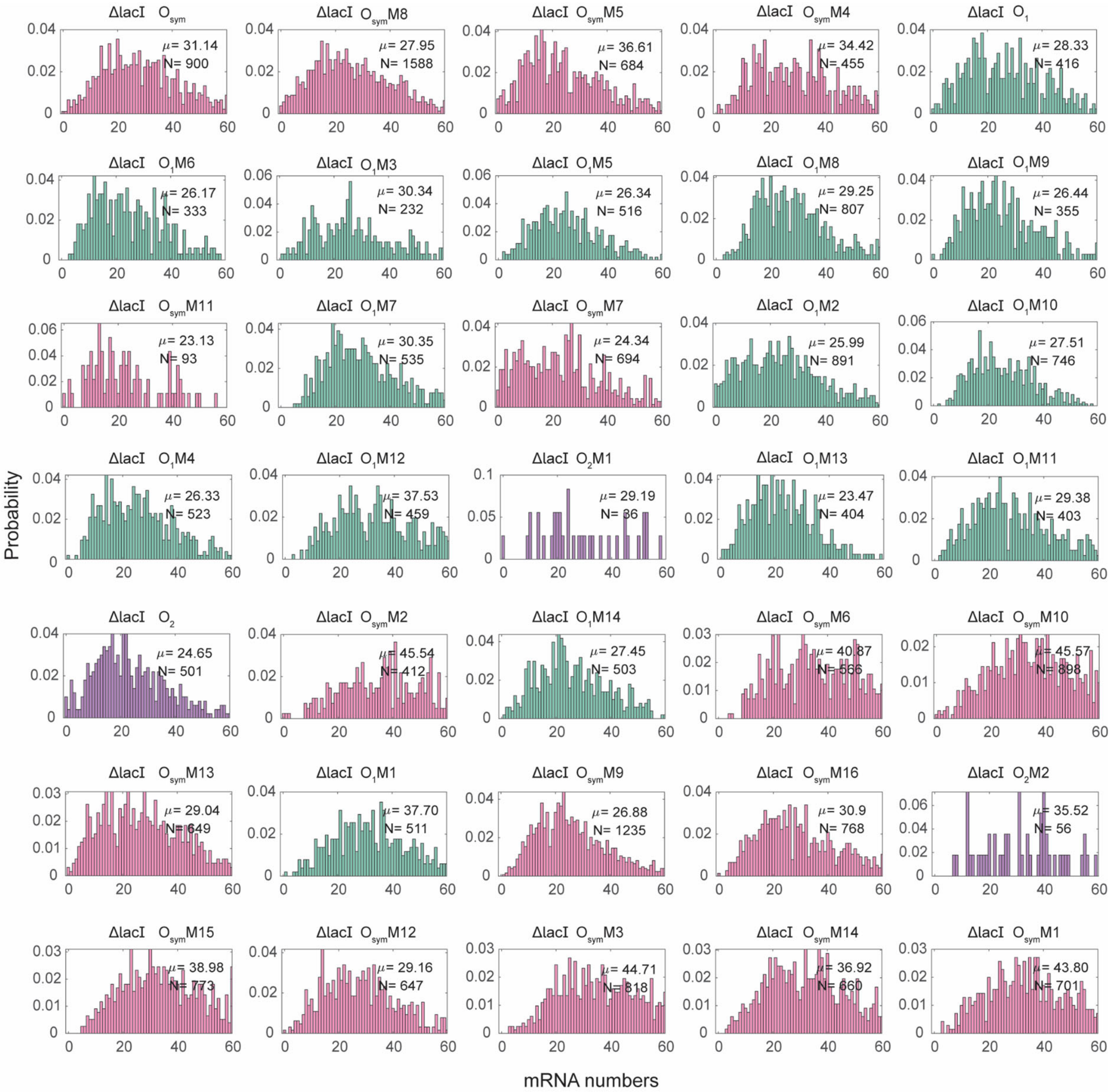

Supplementary Fig.7: Observed mRNA copy number distributions in *ΔlacI* strains, for all operators assayed in this work.

Supplementary Fig.8

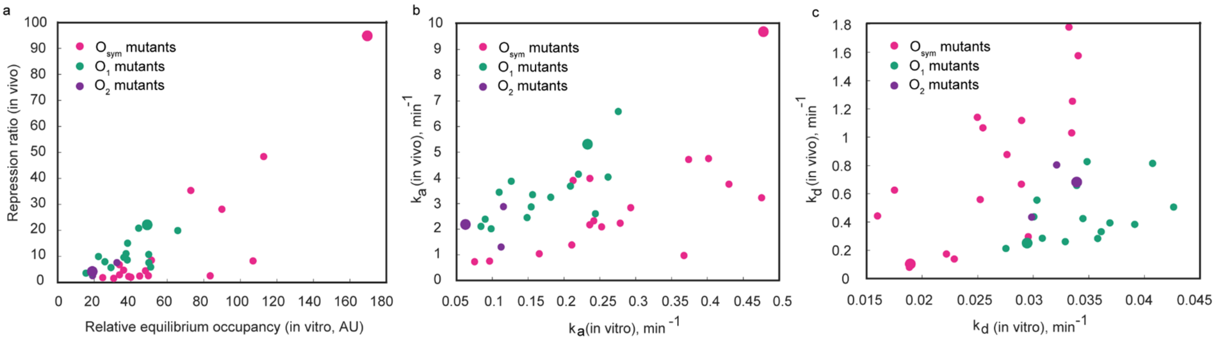

Supplementary Fig. 8: Comparison of in vivo and in vitro data. **a** The repression ratio (defined as the ratio of mRNA expression in the *ΔlacI* strain background to expression in the *wt lacI* strain background) is plotted against the

relative *in vitro* occupancy measured in[1]. As expected, operators with higher occupancy display higher repression ratios. **b** and **c** The association rate k_a and dissociation rate k_d are compared in vivo and in vitro (again using data from[1].). The two measurements are correlated, but O_{sym} mutants generally display slower association and faster dissociation than expected based on i*n vitro* values.

Supplementary Fig. 9

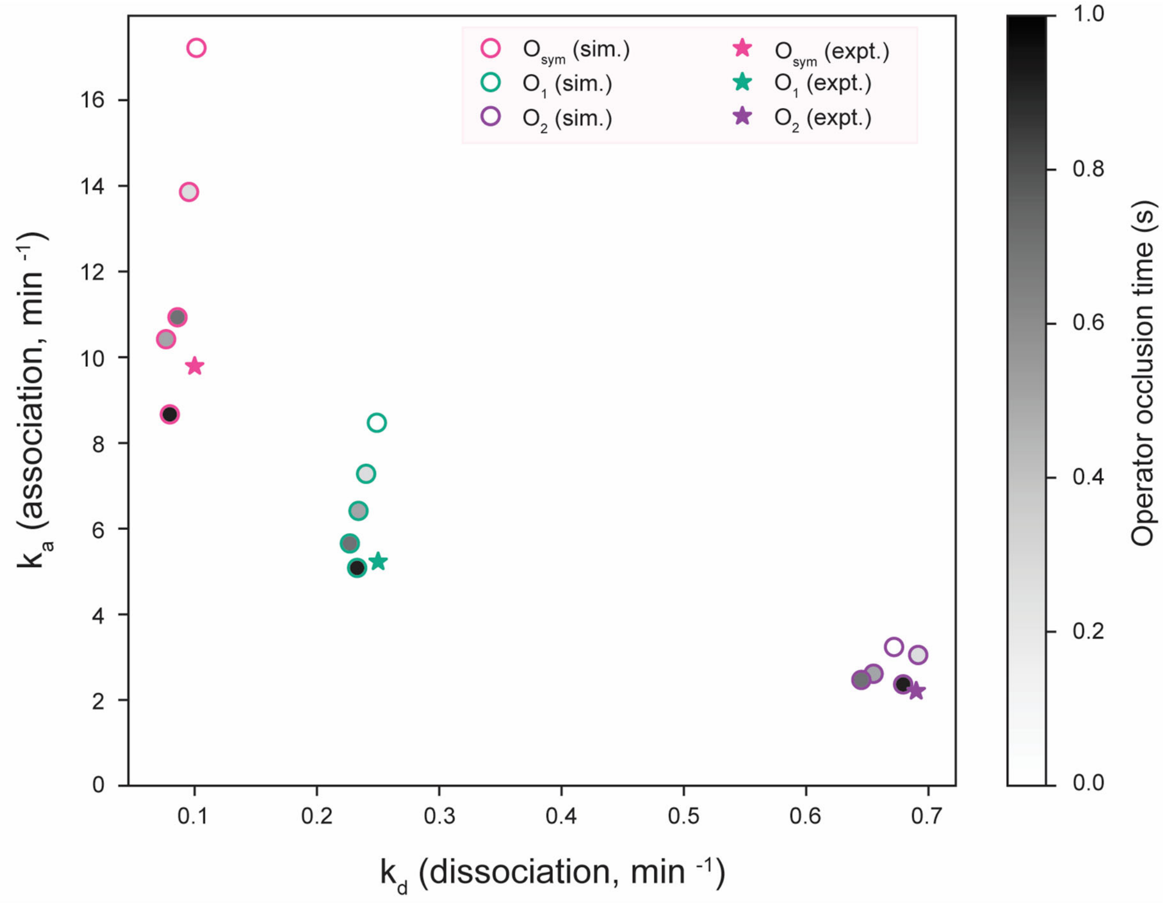

Supplementary Fig. 9: Effect of RNAP occlusion of LacI binding sites on observed association and dissociation rates. While clearing the promoter region, RNAP may temporarily inhibit LacI from binding. This "occlusion time" may in turn affect our calculations of LacI binding kinetics. To estimate the magnitude of this effect, we performed stochastic simulations as in Figure 1, with the modification that, upon production of a transcript, the association rate k_a is set to zero for a time $t_{occlude}$ after the transcription event, in order to reflect the unavailability of the operator for LacI binding. Specifically, for each of the operators O_{sym}, O_1, and O_2, we simulated a population of 20 000 cells for 100 minutes, for occlusion times $t_{occlude}$ = 0, 0.25, 0.5, 0.75, and 1 seconds. At the end of the simulation, the mRNA copy number distribution over the 20 000 cells was computed, and k_a and k_d were estimated from simulated data using the procedure outlined in Figure 2 (circles; grayscale shading in circles reflects $t_{occlude}$ values). For each of the operators, the k_a and k_d values in the $t_{occlude}$=0 case were chosen such that estimated k_a and k_d values in the $t_{occlude}$=1 case would be close to the experimentally determined values (stars). Longer occlusion times lead to lower observed association rates, since LacI must bind in the intervals between transcript production events. Thus, it is possible that the experimentally-determined k_a values reported in this work underestimate the "true" k_a values, but this should not affect the observed anticorrelation between k_a and k_d. In fact, these simulations indicate that the true spread in k_a values may be larger than what we report.

Supplementary Fig. 10

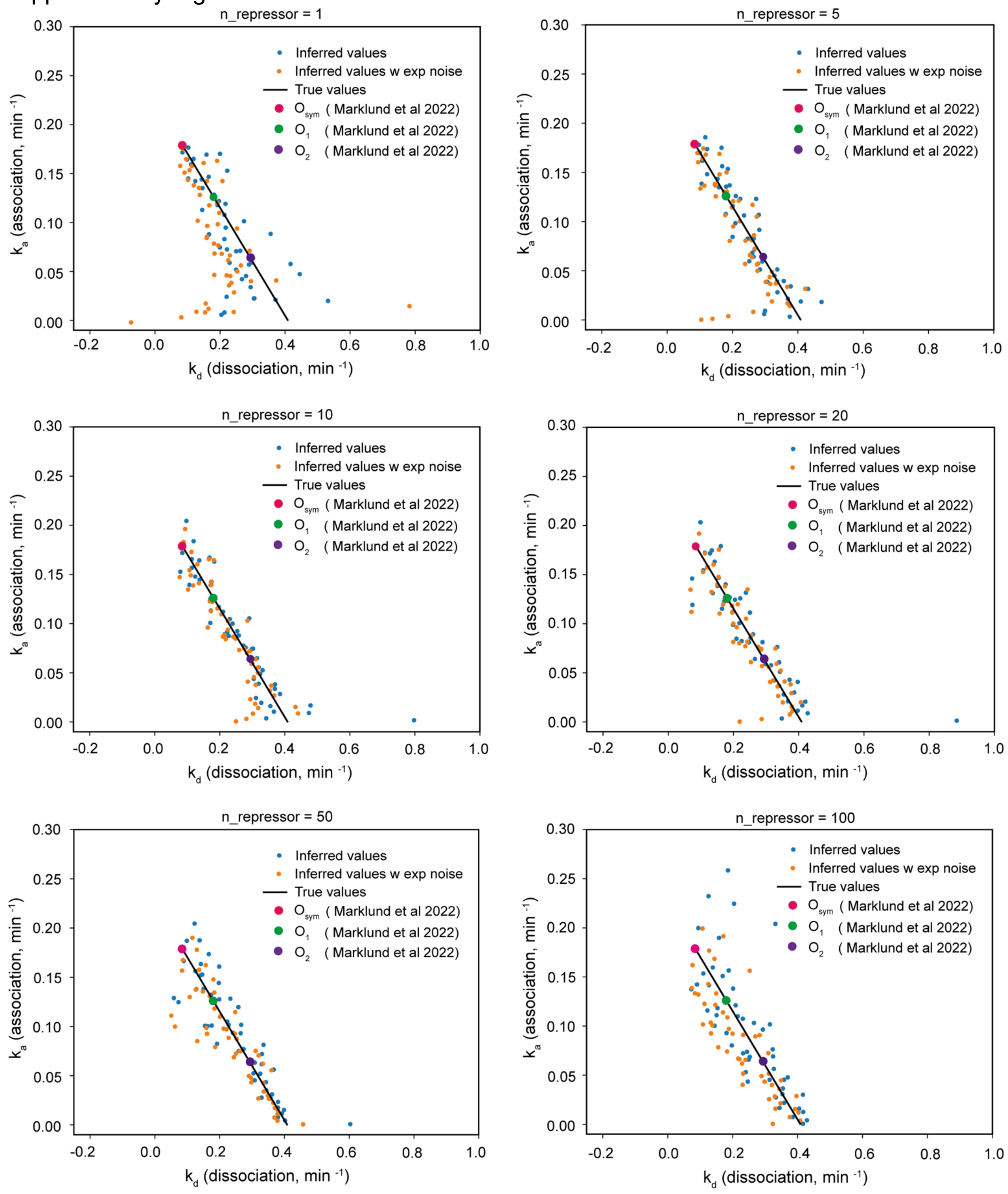

Supplementary Fig. 10: Analysis of simulated data for varying LacI concentrations. In order to assess the feasibility of the project, and to choose an appropriate LacI expression level, a set of 50 simulated operators was created whose association rate per repressor k_a^0 and dissociation rate k_d values were linearly spaced along the line defined in Marklund *et al*[1] (solid black lines, “True values”). In order to capture the effect of varying repressor copy number $n_{repressor}$, the association rate k_a was assumed to be proportional to repressor copy number: $k_a = n_{repressor} \cdot k_a^0$. We examined 6 different repressor copy numbers, varying from 1 to 100 per cell. For a given operator and repressor copy number, the steady-state mRNA copy number distribution was computed numerically, again as described in Sanchez *et al*[2], and a set of 1000 samples (i.e. analogous to performing mRNA FISH on a population of 1000 cells) was drawn from the distribution. The observed mRNA copy numbers in the simulated population were then analyzed according to the procedure in Fig. 2, yielding the “Inferred values” (blue dots). To facilitate comparison between different simulated repressor copy numbers, the association rate per repressor k_a^0 is plotted on the y axis rather than association rate k_a. Note that k_a and k_d values in this figure are based on *in vitro* data and therefore do

not perfectly match *in vivo* estimates from Figs. 2 and 3, although trends are the same. In order to estimate the effects of experimental noise, each mRNA within each simulated cell was assigned a “fluorescence intensity” randomly chosen from a Gaussian distribution with a mean 1 and a standard deviation 0.4. The intensities from all mRNA within a particular cell were summed and rounded to the nearest integer, similarly to how experimental data is analyzed. This data was also analyzed according to the procedure in Fig. 2 (“Inferred values w exp noise”, orange dots), and evidently, this level of variability in the single mRNA intensity did not lead to notably worse inference of LacI kinetics. Inference is most accurate at circa 10 repressors per cell, similar to the wild-type LacI expression[3]. For lower or higher LacI expression, the inferred values are less tightly clustered around the true values. Basal transcription rate r = 22 min^{-1} and mRNA degradation rate γ = 0.8 min^{-1} in these simulations.

Supplementary Table 1: Comparison of parameters for *in vitro* and *in vivo* measurements of LacI kinetics.

Parameter	*in vitro* value[1]	*in vivo* value	Expected change in *in vivo* k_a	Expected change in *in vivo* k_d
LacI concentration	1.7 nM	~10 nM	↑ (higher LacI concentration)	no effect
LacI modifications	Cy3-labeled LacI dimer	None (*wt* protein)	↑ (estimated 2x higher for wt tetramer than than labelled dimer)	no effect (mutations distal to DNA binding domain; Cy3 is small dye)
Salt concentration (sum of Na+ and K+)	30 mM	200-300 mM	unclear; higher salt → less sliding → lower k_a, but too much sliding → locally stuck	↑ (higher salt → less sliding → LacI less likely to rebind after dissociation)
Presence of nonspecific DNA	60 bp per specific site	~5 million bp per specific site	↓ (large amount of nonspecific DNA)	no effect
Other DNA-binding proteins ("roadblocks")	None	Estimated 1 per 36 bp [4]	↓ ("roadblocks" can hinder binding, but mitigated by 3D diffusion?) [5]	possible slight ↓ (more rebinding if roadblocks trap LacI near operator)
DNA topological structure	Little to none (DNA oligos tethered to microarray)	Extensive structure due to NAPs	possible ↓ (DNA topology could locally trap LacI, but mitigated by 3D diffusion?)	possible slight ↓ (more rebinding if DNA topology traps LacI near operator)

Supplementary Table 2: List of strains constructed in the study

S.no	Operator name	Operator Sequence	wt lacI strain number	ΔlacI strain number
1	Osym	AATTGTGAGCGCTCACAATT	EL3853	EL3877
2	O1	AATTGTGAGCGGATAACAATT	EL3854	EL3878
3	O2	GGTTGTTACTCGCTCACATTT	EL3855	EL3879
4	Osym M1	AATTGTGAGCCGTCACAATT	EL3857	EL3867
5	Osym M2	AATTGAGAGCGCTCACAATT	EL3858	EL3868
6	Osym M3	AATTGTGAGCGCTCATAATT	EL3859	EL3869
7	Osym M4	ACTTGTGAGCGCTCACAATT	EL4135	EL4148
8	Osym M5	AATTGTGAGCGCTCACACTT	EL4123	EL4136
9	Osym M6	AATTGTGAGCGCTCGCAATT	EL4124	EL4137
10	Osym M7	AAAAGTGAGCGCTCACAATT	EL4125	EL4138
11	Osym M8	AAATGTGAGCGCTCACAATT	EL4126	EL4139
12	Osym M9	AATTGTGAGCGCTCAAAATT	EL4127	EL4140
13	Osym M10	AATTGTGAGCGCTCACGATT	EL4128	EL4141
14	Osym M11	AATTGTGAGCGTTCACAATT	EL4129	EL4142
15	Osym M12	AATTGTGAGCTCTCACAATT	EL4130	EL4143
16	Osym M13	AATTGTGAGGGCTCACAATT	EL4131	EL4144
17	Osym M14	AATTGTGAGGGGTCACAATT	EL4132	EL4145
18	Osym M15	AATTGTGAGTGCTCACAATT	EL4133	EL4146
19	Osym M16	AATTGTGTGCGCTCACAATT	EL4134	EL4147
20	O1 M1	ATTGTGAGCGGATAGCAATT	EL4160	EL4172
21	O1 M2	AAATGTGAGCGGATAACAATT	EL3862	EL3872
22	O1 M3	AATTGTGAGCGCATAACAATT	EL3863	EL3873
23	O1 M4	ATTGTGAGCGTATAACAATT	EL4149	EL4161
24	O1 M5	ATTGTGAGCGGGTAACAATT	EL4150	EL4162
25	O1 M6	ATTGTGAGCGCATAACAAAT	EL4151	EL4163
26	O1 M7	ATTGTGAGCGAATAACAATT	EL4152	EL4164
27	O1 M8	AATGTGAGCGTATAACAATT	EL4153	EL4165
28	O1 M9	GTTGTGAGCGGATAACAATT	EL4154	EL4166
29	O1 M10	ATTGTGAGCGGATCACAATT	EL4155	EL4167
30	O1 M11	ATTGTGACCGGATAACAATT	EL4156	EL4168
31	O1 M12	CTTGTGAGCGGATAACAATT	EL4157	EL4169
32	O1 M13	ATTGTGAGCGGATCACAATC	EL4158	EL4170
33	O1 M14	ATTGTGAGCGGACAACAATT	EL4159	EL4171
34	O2 M1	CGTTGTTACACGCTCACATTT	EL3866	EL3876
35	O2 M2	GGTTGTTACACGCTCACATTT	EL3865	EL3875

Supplementary Table 3: List of oligos used in the study

s.no	Oligos name	Oligo sequence (5'--->3')
1	Del_LacI_Direx-F	ggtggtgaatgtgaaaccagtaacgttatacgatgtcgcacaggtttcccgactggtgtaggctggagctgcttc
2	cat_mid-R	gccgacatggaagccatcac
3	cat_mid-F	cgacgatttccggcagtttc
4	Del_LacI_Direx-R	gcgttgcgctcactgcccgctttccagtcgggaaacctgtgcgacatcgtataagtgtaggctggagctgcttc
5	ygaY_HA_F	ccacaaatgaaatcagaaacccacctcaactacccacagttgttgggccctgaaaaatgcatagtgc
6	ygaY_HA_R	acgcgcgggtacgcagaattttatcgctgataaacatactgaaaacggaaggaccaaaacgaaaaaag
7	mVenusNB_RT_F	tgcgagatacccagatcata
8	mVenusNB_RT_R	cttgtagttcccgtcatcttt
9	rrsA_RT_F	ctcttgccatcggatgtgccca
10	rrsA_RT_R	cagtgtggctggtcatcctctca

Supplementary Table 4: List of FISH probe sequences used in this study

Transcript	name	sequence (5' ---> 3')	Position	GC percentage	Conjugant
mVenusNB	mVenus1	aagttcttctcctttactca	2	35.00%	Cy5
mVenusNB	mVenus2	gaattgggacaactccagtg	24	50.00%	Cy5
mVenusNB	mVenus3	cccattaacatcaccatcta	53	40.00%	Cy5
mVenusNB	mVenus4	cctctccactgacagaaaat	78	45.00%	Cy5
mVenusNB	mVenus5	gtaagttttccgtatgttgc	109	40.00%	Cy5
mVenusNB	mVenus6	gttttccagtagtgcaaatc	138	40.00%	Cy5
mVenusNB	mVenus7	aagtgttggccatggaacag	161	50.00%	Cy5
mVenusNB	mVenus8	caccataacccaaagtagtg	183	45.00%	Cy5
mVenusNB	mVenus9	gggtatctcgcaaagcattg	205	50.00%	Cy5
mVenusNB	mVenus10	gtcatgctgtttcatatgat	227	35.00%	Cy5
mVenusNB	mVenus11	cgggcatggcactcttgaaa	249	55.00%	Cy5
mVenusNB	mVenus12	gttctttcctgtacataacc	271	40.00%	Cy5
mVenusNB	mVenus13	gttcccgtcatctttgaaaa	296	40.00%	Cy5
mVenusNB	mVenus14	tgacttcagcacgtgtcttg	318	50.00%	Cy5
mVenusNB	mVenus15	taacaagggtatcaccttca	342	40.00%	Cy5
mVenusNB	mVenus16	ataccttttaactcgattct	364	30.00%	Cy5
mVenusNB	mVenus17	gaatgtttccatcttcttta	390	30.00%	Cy5
mVenusNB	mVenus18	ttgtattccaatttgtgtcc	412	35.00%	Cy5
mVenusNB	mVenus19	gccgtgatgtatacattgtg	442	45.00%	Cy5
mVenusNB	mVenus20	gattccattcttttgtttgt	464	30.00%	Cy5
mVenusNB	mVenus21	gtctaattttgaagttcgct	486	35.00%	Cy5
mVenusNB	mVenus22	aacacctccatcttcaatgt	509	40.00%	Cy5
mVenusNB	mVenus23	gttgataatggtctgctagt	531	40.00%	Cy5
mVenusNB	mVenus24	ccatcgccaattggagtatt	553	45.00%	Cy5
mVenusNB	mVenus25	ggttgtctggtaaaaggaca	576	45.00%	Cy5
mVenusNB	mVenus26	cttagattggtaggacaggt	599	45.00%	Cy5
mVenusNB	mVenus27	tttcgttgggatctttcgaa	621	40.00%	Cy5
mVenusNB	mVenus28	agaaggaccatgtgatctct	643	45.00%	Cy5
mVenusNB	mVenus29	atcccagcagctgttacaaa	667	45.00%	Cy5
mVenusNB	mVenus30	tatagttcatccatgccatg	691	40.00%	Cy5

Supplementary References

1. Marklund, E. *et al.* Sequence specificity in DNA binding is mainly governed by association. *Science* 375, 442–445 (2022).

2. Sanchez, A., Garcia, H. G., Jones, D., Phillips, R. & Kondev, J. Effect of Promoter Architecture on the Cell-to-Cell Variability in Gene Expression. *PLOS Comput. Biol.* **7**, e1001100 (2011).

3. Xie, X. S., Choi, P. J., Li, G.-W., Lee, N. K. & Lia, G. Single-Molecule Approach to Molecular Biology in Living Bacterial Cells. Annu. Rev. Biophys. 37, 417–444 (2008).

4. Li, G.W. et al. Quantifying absolute protein synthesis rates reveals principles underlying allocation of cellular resources. Cell. Apr 24;157(3):624-35 (2014).

5. Hammar, P. et al. Direct measurement of transcription factor dissociation excludes a simple operator occupancy model for gene regulation. Nat. Genet. 46, 405–408 (2014).
