# Supplementary material for: Anti-correlation of LacI association and dissociation rates observed in living cells
Source: Nat Commun. 2025 Jan 17;16:764. doi: 10.1038/s41467-025-56053-z (PMC11748676; doi:10.1038/s41467-025-56053-z)
Supplement: Supplementary file 2 — Reporting Summary [file 41467_2025_56053_MOESM2_ESM.pdf]

nature portfolio

Corresponding author(s): Daniel Jones and Johan Elf

Last updated by author(s): Jan 1, 2025

Reporting Summary

Nature Portfolio wishes to improve the reproducibility of the work that we publish. This form provides structure for consistency and transparency in reporting. For further information on Nature Portfolio policies, see our Editorial Policies and the Editorial Policy Checklist.

Statistics

For all statistical analyses, confirm that the following items are present in the figure legend, table legend, main text, or Methods section.

n/a	Confirmed	
[ ]	[x]	The exact sample size (*n*) for each experimental group/condition, given as a discrete number and unit of measurement
[ ]	[x]	A statement on whether measurements were taken from distinct samples or whether the same sample was measured repeatedly
[x]	[ ]	The statistical test(s) used AND whether they are one- or two-sided<br>*Only common tests should be described solely by name; describe more complex techniques in the Methods section.*
[x]	[ ]	A description of all covariates tested
[x]	[ ]	A description of any assumptions or corrections, such as tests of normality and adjustment for multiple comparisons
[ ]	[x]	A full description of the statistical parameters including central tendency (e.g. means) or other basic estimates (e.g. regression coefficient) AND variation (e.g. standard deviation) or associated estimates of uncertainty (e.g. confidence intervals)
[x]	[ ]	For null hypothesis testing, the test statistic (e.g. *F*, *t*, *r*) with confidence intervals, effect sizes, degrees of freedom and *P* value noted<br>*Give P values as exact values whenever suitable.*
[x]	[ ]	For Bayesian analysis, information on the choice of priors and Markov chain Monte Carlo settings
[x]	[ ]	For hierarchical and complex designs, identification of the appropriate level for tests and full reporting of outcomes
[x]	[ ]	Estimates of effect sizes (e.g. Cohen's *d*, Pearson's *r*), indicating how they were calculated

Our web collection on statistics for biologists contains articles on many of the points above.

Software and code

Policy information about availability of computer code

Data collection	High-throughput microscopy experiments were acquired Micro-manager 1.4 version. qPCR measurements were performed using the Applied Biosystems, StepOne Software v2.3
Data analysis	We use custom image processing Matlab (2020a) code with deep learning models. The code and models are also attached to the submission.

For manuscripts utilizing custom algorithms or software that are central to the research but not yet described in published literature, software must be made available to editors and reviewers. We strongly encourage code deposition in a community repository (e.g. GitHub). See the Nature Portfolio guidelines for submitting code & software for further information.

Data

Policy information about availability of data

All manuscripts must include a data availability statement. This statement should provide the following information, where applicable:

- Accession codes, unique identifiers, or web links for publicly available datasets
- A description of any restrictions on data availability
- For clinical datasets or third party data, please ensure that the statement adheres to our policy

Raw microscopy data is publicly available at https://doi.org/10.17044/scilifelab.26425576.v1

Research involving human participants, their data, or biological material

Policy information about studies with human participants or human data. See also policy information about sex, gender (identity/presentation), and sexual orientation and race, ethnicity and racism.

Reporting on sex and gender	NA
Reporting on race, ethnicity, or other socially relevant groupings	NA
Population characteristics	NA
Recruitment	NA
Ethics oversight	NA

Note that full information on the approval of the study protocol must also be provided in the manuscript.

Field-specific reporting

Please select the one below that is the best fit for your research. If you are not sure, read the appropriate sections before making your selection.

[x] Life sciences [ ] Behavioural & social sciences [ ] Ecological, evolutionary & environmental sciences

For a reference copy of the document with all sections, see nature.com/documents/nr-reporting-summary-flat.pdf

Life sciences study design

All studies must disclose on these points even when the disclosure is negative.

Sample size	The required sample sizes (number of individual cells per operator) were estimated by simulation. In Supplementary Fig 10, we found that 1000 cells was sufficient to accurately determine LacI association and dissociation rates, and consequently aimed for at least 1000 cells per operator spread across at least two biological replicates. Ultimately we ended up with between 1445 and 5415 cells per operator.
Data exclusions	Data that produced cell segmentation errors were excluded from analysis based on cell width and area. These data are typically a small fraction. Cells with lengths outside of a defined range were excluded from analysis, in order to ensure that analyzed cells contain a single copy of the expression construct (as described in main text).
Replication	mRNA FISH data for wt LacI strains consists of at least 3 biological replicates on different days. mRNA FISH data for Del LacI strains consists of at least 2 biological replicates on different days. qPCR data consists of 3 biological replicates on different days. Protein data consists of at least 2 biological replicates. These results were reproducible and consistent between the replicates.
Randomization	NA, since we compared different sequence mutants of LacI binding sites.
Blinding	Blinding was not possible because each LacI binding site sequence needed to be analyzed individually.

Reporting for specific materials, systems and methods

We require information from authors about some types of materials, experimental systems and methods used in many studies. Here, indicate whether each material, system or method listed is relevant to your study. If you are not sure if a list item applies to your research, read the appropriate section before selecting a response.

Materials & experimental systems

n/a	Involved in the study	
[x]	[ ]	Antibodies
[x]	[ ]	Eukaryotic cell lines
[x]	[ ]	Palaeontology and archaeology
[x]	[ ]	Animals and other organisms
[x]	[ ]	Clinical data
[x]	[ ]	Dual use research of concern
[x]	[ ]	Plants

Methods

n/a	Involved in the study	
[x]	[ ]	ChIP-seq
[x]	[ ]	Flow cytometry
[x]	[ ]	MRI-based neuroimaging

Plants

Seed stocks	NA
Novel plant genotypes	NA
Authentication	NA
